# Parametrically enhanced interactions and nonreciprocal bath dynamics in a photon-pressure Kerr amplifier

**DOI:** 10.1126/sciadv.abq1690

**Published:** 2022-08-26

**Authors:** Ines Corveira Rodrigues, Gary Alexander Steele, Daniel Bothner

**Affiliations:** ^1^ Kavli Institute of Nanoscience, Delft University of Technology, PO Box 5046, 2600 GA Delft, Netherlands.; ^2^ Department of Physics, ETH Zürich, Zurich, Switzerland.; ^3^ Physikalisches Institut and Center for Quantum Science in LISA^+^, Universität Tübingen, 72076 Tübingen, Germany.

## Abstract

Photon-pressure coupling between two superconducting circuits is a promising platform for investigating radiation-pressure coupling in distinct parameter regimes and for the development of radio-frequency (RF) quantum photonics and quantum-limited RF sensing. Here, we implement photon-pressure coupling between two superconducting circuits, one of which can be operated as a parametric amplifier. We demonstrate a Kerr-based enhancement of the photon-pressure single-photon coupling rate and an increase of the cooperativity by one order of magnitude in the amplifier regime. In addition, we observe that the intracavity amplification reduces the measurement imprecision of RF signal detection. Last, we demonstrate that RF mode sideband cooling is unexpectedly not limited to the effective amplifier mode temperature arising from quantum noise amplification, which we interpret in the context of nonreciprocal heat transfer between the two circuits. Our results demonstrate how Kerr amplification can be used as resource for enhanced photon-pressure systems and Kerr cavity optomechanics.

## Teaser

Heat flowing upstream: strong driving of a nonlinear circuit enables thermal excitations to flow from cold to hot.

## INTRODUCTION

Photon-pressure and radiation-pressure coupled oscillators, where the amplitude of one oscillator modulates the resonance frequency of the second, have enabled a large variety of groundbreaking experiments in the recent decades. In cavity optomechanics (*1*), this type of coupling has been used for unprecedented precision in the detection and control of mechanical displacement (*2*–*7*), to generate entanglement between two mechanical oscillators (*8*, *9*), and to realize nonreciprocal signal processing (*10*–*12*), parametric microwave amplification (*13*–*15*), frequency conversion (*16*–*18*), and the generation of entangled radiation (*19*), to name just a few of the highlights. More recently, the implementation of photon-pressure coupling between two superconducting circuits has attracted a lot of attention (*20*–*23*). Notably, within a short period of time, the strong-coupling regime, the quantum-coherent regime, and the sideband cooling of a hot radio-frequency (RF) circuit into its quantum ground state have been achieved (*24*, *25*). These recent results open the door for quantum-limited photon-pressure microwave technologies, RF quantum photonics, and quantum-enhanced dark matter axion detection at low-energy scales (*26*–*28*) and for new approaches in circuit-based quantum information processing in terms of fault-tolerant bosonic codes (*29*).

Photon-pressure coupled circuits use a superconducting quantum interference device (SQUID) as a key coupling element, similar to flux-mediated optomechanics (*30*–*34*), and therefore, these platforms naturally come with Kerr cavities due to the Josephson nonlinearity of the SQUID inductance. Most experimental and theoretical works on optomechanical and photon-pressure systems have considered only the case of photon-pressure coupled linear oscillators, but lately, there has been growing interest in Kerr-like nonlinearities in photon-pressure interacting systems (*35*–*42*). Kerr nonlinearities in superconducting circuits are already extremely useful resources for cat-state quantum computation (*43*); for quantum-limited signal processing and detection by means of stand-alone Josephson parametric amplifiers (JPAs), circulators, and converters (*44*–*48*); and for Josephson metamaterials (*49*–*51*). Adding these exciting functionalities to photon-pressure coupled and optomechanical systems constitutes therefore a highly promising approach for enhanced quantum sensing devices and novel photon control schemes.

Here, we report photon-pressure coupling between a superconducting RF circuit and a strongly driven superconducting Kerr cavity, operated as a parametric amplifier. As well known from previous work (*42*, *52*–*55*), by strongly driving the high-frequency (HF) SQUID cavity of our system, we can activate a four-wave mixing process and obtain an effective signal-idler double-mode cavity, here reaching up to ∼12 dB of intracavity gain. Furthermore, by using an additional pump tone applied to the red sideband of the signal-mode resonance, we simultaneously switch on the photon-pressure coupling between this quasi-mode and the RF circuit.

We observe that the strong parametric drive enhances the single-photon coupling rate between the circuits, which, in combination with further enhancement effects, eventually leads to a more than 10-fold increment in effective cooperativity. Using the device as an RF thermal noise upconverter, we find that the output noise is accordingly amplified by the intrinsic Josephson amplification, which is potentially interesting for enhanced detection of weak RF signals. Last, we observe that sideband cooling of the RF mode is not limited to the effective photon occupation of the quantum-heated amplifier mode and that the cooling tone is increasing the population imbalance between the two modes instead of reducing it. Our results using a driven Kerr cavity disclose physical phenomena that have not yet been observed or described in standard radiation-pressure systems and that are potentially useful for sensing of weak RF signals, microwave signal processing, and Kerr optomechanical systems.

## RESULTS

### Device and photon-pressure coupling

**Fig. 1.**
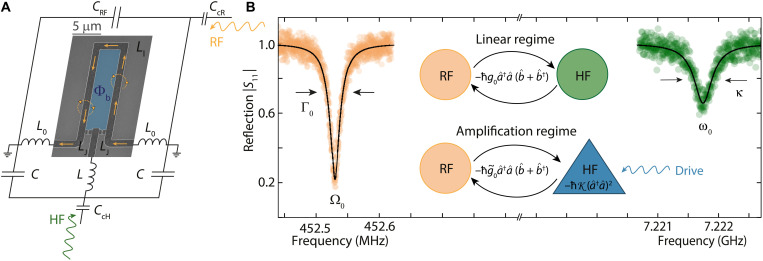
Photon-pressure coupling between an RF LC circuit and a parametric amplifier SQUID cavity. (**A**) Circuit schematic with an embedded scanning electron microscopy image of the SQUID. The HF mode consists of the linear inductors *L* and *L*_0_, the capacitor *C*, and the Josephson inductances *L*_J_. The RF mode consists of the capacitor *C*_RF_ and the linear inductors *L*_0_ and *L*_l_. Each mode is capacitively coupled to an individual feedline for driving and readout by means of a coupling capacitor *C*_cR_ and *C*_cH_. The SQUID in the center of the circuit is biased with an external coil to a magnetic flux Φ_b_, and any current from the RF mode flowing through the SQUID, indicated as yellow arrows, will add additional fluctuating flux Φ_RF_. Both the bias flux and the RF flux will change the inductance of the Josephson junctions in the SQUID *L*_J_ (Φ). (**B**) The reflection response of the RF and HF mode in their corresponding frequency ranges and measured via their individual feedlines, respectively. The RF mode displays a resonance frequency Ω_0_ = 2π∙452.53 MHz and a total linewidth Γ_0_ = 2π · 45 kHz. For the HF mode, we get ω_0_ = 2π · 7.2218 GHz and the linewidth κ = 2π · 400 kHz. Both resonance frequency and linewidth depend on the flux bias, and, here, Φ_b_/Φ_0_ = 0.48. The inset schematically shows the two photon-pressure operation modes implemented here. In the linear regime, the RF circuit is coupled via photon-pressure to a linear HF cavity; in the amplification regime, the RF mode is coupled to a Kerr parametric amplifier. The amplification regime is activated by a near-resonant strong HF cavity drive. The single-photon coupling rates are given by *g*_0_ and ${\tilde{g}}_{0}=g_{0}+\frac{g_{K}}{2}{\hat{a}}^{†}\hat{a}$, respectively.

Our device combines a superconducting RF LC circuit with a superconducting microwave SQUID cavity in a galvanic coupling architecture (*25*); cf. Fig. 1A. The circuit resonance frequencies are ω_0_ = 2π · 7.222 GHz for the HF mode and Ω_0_ = 2π · 452.5 MHz for the RF mode; cf. Fig. 1B. We note that, although it is the same device used in (*25*), its properties are not identical as it has gone through several warmups and cooldowns and has been repositioned and recabled inside the dilution refrigerator, and it is operated at a different bias flux point now. Details on the device fabrication and the measurement setup can be found in Materials and Methods and in notes S1 and S2.

At the heart of the device is a nanobridge-based SQUID, which translates the magnetic flux connected to oscillating currents in the RF inductor into resonance frequency modulations of the HF circuit.

To first order and without taking into account the nonlinearity of the Josephson nanobridges, the Hamiltonian of the undriven system is given by

$${\hat{H}}_{\text{lin}}=\hbar \omega_{0}{\hat{a}}^{†}\hat{a}+\hbar \Omega_{0}{\hat{b}}^{†}\hat{b}+\hbar g_{0}{\hat{a}}^{†}\hat{a}(\hat{b}+{\hat{b}}^{†})$$ (1)

where the photon-pressure single-photon coupling rate

$$g_{0}=\frac{\partial \omega_{0}}{\partial \Phi }\Phi_{\text{zpf}}$$ (2)

is given by the flux responsivity of the HF mode resonance frequency ∂ω_0_/∂Φ and the effective zero-point RF flux Φ_zpf_ ≈ 635 μΦ_0_ coupling into the SQUID loop; cf. also note S3. Note that, here, the annihilation (creation) operators $\hat{a},\hat{b}\;({\hat{a}}^{†},{\hat{b}}^{†})$ refer to a change in photon excitations of the HF and RF circuit, respectively, and that the RF-induced flux $\hat{\Phi }=\Phi_{\text{zpf}}(\hat{b}+{\hat{b}}^{†})$ threading the SQUID is analogous to the displacement of a mechanical resonator in an optomechanical system.

When the Kerr nonlinearity of the Josephson junctions is taken into account, the Hamiltonian $\hat{H}={\hat{H}}_{\text{lin}}+{\hat{H}}_{\text{Kerr}}$ is extended with the Kerr terms (*37*)

$${\hat{H}}_{\text{Kerr}}=\frac{\hbar K}{2}{\left({\hat{a}}^{†}\hat{a}\right)}^{2}+\frac{\hbar g_{K}}{2}{\left({\hat{a}}^{†}\hat{a}\right)}^{2}(\hat{b}+{\hat{b}}^{†})$$ (3)

where the Kerr-related photon-pressure coupling constant is given by

$$g_{K}=\frac{\partial K}{\partial \Phi }\Phi_{\text{zpf}}$$ (4)

Here, we omitted the nonlinearity of the RF circuit as it is extremely small with K_RF_ ∼ − 2π · 1 Hz. For the HF circuit, the Kerr constant $K=-\frac{e^{2}}{2\hbar C_{\text{HF}}}\frac{L_{J}^{3}}{L_{\text{HF}}^{3}}$ depends on the bias flux via the inductance ratio and is on the order of K ∼ − 2π · 5 kHz.

The interaction part of the Hamiltonian is therefore given by

$${\hat{H}}_{\text{int}}=\hbar g_{0}{\hat{a}}^{†}\hat{a}(\hat{b}+{\hat{b}}^{†})+\frac{\hbar g_{K}}{2}{\left({\hat{a}}^{†}\hat{a}\right)}^{2}(\hat{b}+{\hat{b}}^{†})$$ (5)

In the following section, we will investigate the linearized dynamics of this system under strong near-resonant driving and for the case of a combination of near-resonant driving and additional photon-pressure red sideband pumping.

### Kerr amplifier quasi-modes

**Fig. 2.**
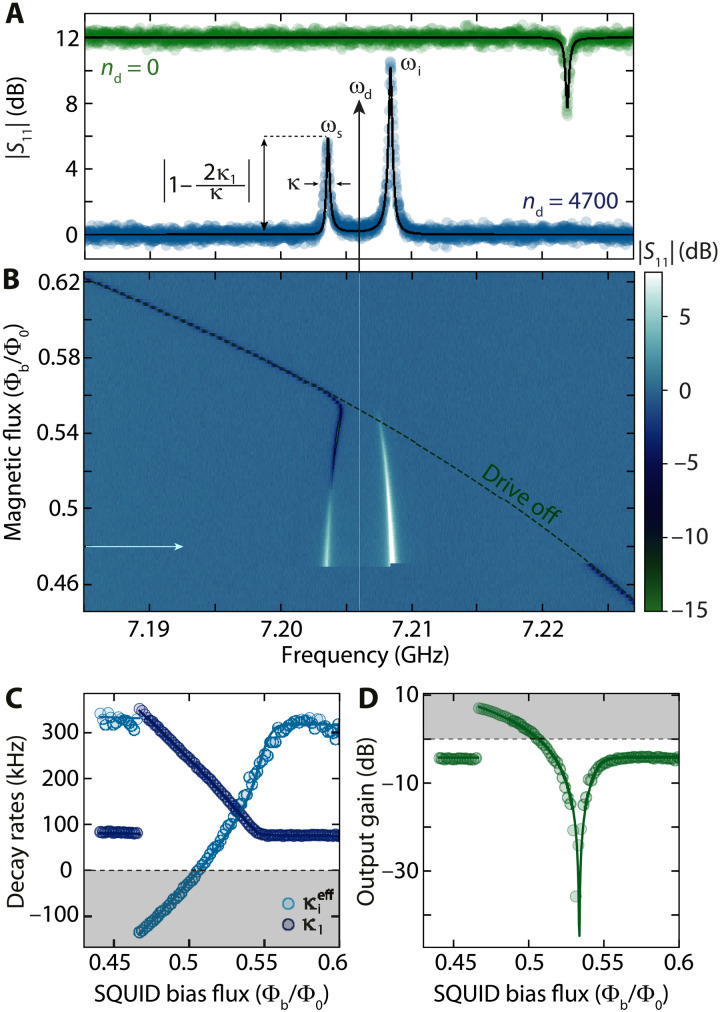
Observation of parametric gain in a strongly driven photon-pressure Kerr cavity. (**A**) Probe-tone reflection off the HF SQUID cavity *S*_11_ with (blue) and without (green) a strong drive at ω_d_. The undriven response labeled *n*_d_ = 0 is offset by +12 dB for clarity. While the undriven cavity displays a single absorption resonance at ω_0_, the driven state (*n*_d_ = 4700) exhibits a double resonance with output gain in both quasi-modes. The signal- and idler-mode resonance frequencies are ω_s_ and ω_i_, respectively. Circles are data; lines are fits. (**B**) Color-coded reflection *S*_11_ in the presence of the strong drive at ω_d_ during a resonance frequency upsweep, displaying the continuous emergence of the double-mode response of the linescan shown in (A) (position indicated by horizontal arrow). The undriven resonance frequency ω_0_(Φ_b_) is indicated as a dashed line. From a fit to the signal mode of each line with a single linear cavity response, i.e., Eq. 8 with κ_2_ = 0, we obtain the apparent external and internal decay rates κ_1_ and $\kappa_{i}^{\text{eff}}=\kappa -\kappa_{1}$, plotted as circles in (**C**). Lines show theoretical calculations based on the full driven Kerr model (see notes S4, S5, and S7), including drive-saturating two-level systems. The intracavity Josephson gain at the amplifier signal resonance is given by $G_{s}=\kappa_{1}/\kappa_{e}$, and the gray shaded area indicates the regime of $\kappa_{i}^{\text{eff}}<0$, i.e., of output gain ***>***1. In (**D**), we show the corresponding output gain at ω = ω_s_, indicating that the effective coupling between the cavity and its readout feedline can be continuously changed using the drive tone, reaching, for instance, the regime of critically coupled at the point where the output gain is lowest (i.e., $\kappa_{i}^{\text{eff}}=\kappa_{1}$). Circles are extracted from data; line is the theoretical prediction.

For a strong near-resonant drive, the dynamics of the HF cavity with respect to a small additional probe field is captured by that of current-pumped JPA. In contrast to usual JPA experiments, however, we operate the amplifier in the high-amplitude state far beyond its bifurcation point and work with a small linewidth cavity κ ∼ 2π · 250 kHz in the undercoupled regime. By doing so, we ensure that the strong drive tone is far detuned from the (phase-insensitive) amplifier mode of interest and that it will not interfere with the photon-pressure interaction in the presence of an additional sideband pump later by coherently driving the RF mode. We prepare the SQUID cavity in this state by using a fixed-frequency drive tone at ω_d_ and by moving the HF cavity resonance frequency ω_0_ from lower to higher frequencies through ω_d_ by means of the SQUID flux bias Φ_b_; cf. Fig. 2. Corresponding data for a flux sweep in the opposite direction can be found in note S6.

The drive-induced modification of the cavity susceptibility leads to several effects regarding the cavity response to an additional probe tone. First, the resonance frequency of the driven mode ω_s_ deviates considerably from the undriven case (dashed line in Fig. 2B) and even tends to shift to lower frequencies with decreasing flux. The reason behind this is the nonlinear frequency shift due to an increasing intracavity drive photon number, which is compensating the flux shift (*42*). Second, we observe that the intracavity Josephson gain turns the resonance absorption dip into a net gain peak, translating a clear change in the effective coupling between the cavity and its feedline. Last, as theoretically and experimentally explored in previous systems (*42*, *52*–*55*), we also observe the emergence of a second peak in the spectral response due to a phenomenon that one can describe as “idler resonance.” Here, the probe tone image frequency, i.e., the frequency of the idler photons generated by nonlinear mixing from the drive and the probe, becomes resonant with the cavity mode (*55*). In this regime, we observe output field gain at the idler resonance and the cavity exhibits an internal feedback locking mechanism that has been used to stabilize the cavity against external flux noise in a related system (*42*). In this experimental situation, the photon-pressure coupling can be neglected to first order, and the linearized probe-tone response is given by

$$S_{11}(\Omega )=1-\kappa_{e}\chi_{G}(\Omega )$$ (6)

with the driven susceptibility

$$\chi_{G}(\Omega )=\frac{\chi_{p}(\Omega )}{1-K^{2}n_{d}^{2}\chi_{p}(\Omega )\chi_{p}^{*}(-\Omega )}$$ (7)

Here, κ_e_ ∼ 2π · 80 kHz is the external coupling rate, *n*_d_ is the intracavity drive photon number, Ω is the probe-tone frequency with respect to ω_d_, and $\chi_{p}^{-1}=\kappa /2+i(\Delta_{d}+2Kn_{d}+\Omega )$ with Δ_d_ = ω_d_ − ω_0_.

To obtain additional intuitive insight, the reflection can also be approximated by a combination of two Kerr-modified conventional modes

$$S_{11}(\Omega )=1-\frac{\kappa_{1}}{\frac{\kappa }{2}+i(\Omega -\Omega_{s})}-\frac{\kappa_{2}}{\frac{\kappa }{2}+i(\Omega -\Omega_{i})}$$ (8)

using the signal and idler mode resonance frequencies Ω_s, i_, the apparent external linewidths κ_1_ = G_s_κ_e_, κ_2_ = G_i_κ_e_, and the intracavity Josephson gain

$$G=\frac{\Omega -\Delta_{d}+2Kn_{d}}{2\Omega }$$ (9)

at the signal and idler mode resonance frequencies G_s, i_ = G(Ω_s, i_). The maximum intracavity Josephson gain for the signal mode is then given by G_s_ = κ_1_/κ_e_ ∼ 12 dB (cf. Fig. 2C), leading to the observed output gain of $G_{\text{out}}=1-\frac{2\kappa_{e}}{\kappa }G_{s}∼6\;$dB (the remaining ∼6 dB of the intracavity gain is required to overcome the internal losses of the undercoupled cavity). Both Josephson gain and effective linewidths of the signal resonance are well captured by the theoretical model (cf. Fig. 2, C and D), if we take the effect of saturating two-level systems into account (*56*), which reduces the total mode linewidth with drive photon number *n*_d_ (for more details, see notes S4 and S6). Note that, when treating the signal resonance as a usual mode, the increasing drive photon number and Josephson gain, respectively, also induce a transition from an undercoupled to a critically coupled to an overcoupled cavity and lastly to a cavity with a negative internal linewidth displaying a net output gain; cf. Fig. 2, C and D. This can be described by a change in the magnitude of the ratio between the effective external and internal linewidths of the driven system $|\kappa_{1}/\kappa_{i}^{\text{eff}}|$ that goes from <1 (undercoupled) to >1 (overcoupled). Note that the total decay rate κ is not affected by the amplification process. Last, this drive-tunable external coupling is advantageous not only to realize nondegenerate parametric amplifiers (*45*) but also for the engineering of tunable microwave attenuators (*46*).

For the remainder of this paper, we will always work at the flux point Φ_b_/Φ_0_ ≈ 0.48, which is indicated by a horizontal arrow in Fig. 2B. In note S9, we also present and discuss a dataset from a different flux point Φ_b_/Φ_0_ ≈ 0.52 with a smaller Josephson gain.

### Parametrically enhanced interaction in a Kerr amplifier

**Fig. 3.**
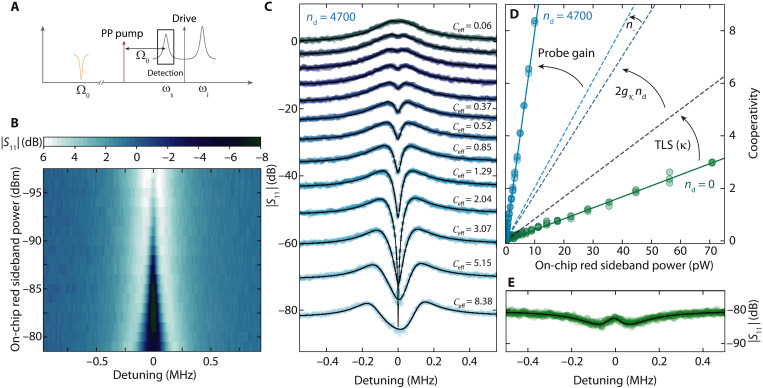
Parametrically enhanced photon-pressure interaction by internal Kerr amplification. (**A**) Experimental protocol for photon-pressure coupling in the high-gain regime. By using a strong drive tone at ω_d_, the HF mode is prepared in the regime where both quasi-modes show output gain (*n*_d_ = 4700, Φ_b_/Φ_0_ = 0.48). An additional photon-pressure pump tone (PP pump) is applied at ω_p_ ≈ ω_s_ − Ω_0_. A third, weak probe tone around ω ≈ ω_s_ detects *S*_11_. (**B**) Color-coded HF reflection *S*_11_ versus detuning from ω_s_ and photon-pressure pump power. Individual linescans are shown in (**C**). Circles are data; lines are the result of theoretical calculations; cf. note S8. The slight peak-height asymmetry for the largest photon-pressure pump powers originates from frequency-dependent Josephson gain. From fits with the effective conventional-mode model discussed in the context of Fig. 2, we obtain the effective cooperativity $C_{\text{eff}}=4g_{\text{eff}}^{2}/({\kappa \Gamma }_{0})=4g_{s}n_{-}{\left(g_{0}+2g_{K}n_{d}\right)}^{2}/({\kappa \Gamma }_{0})$ for each power. The result is shown in (**D**) (blue circles) in direct comparison with the cooperativity for *n*_d_ = 0 and $G_{s}=1$, respectively (green circles). The cooperativity for *n*_d_ = 4700 is enhanced by more than one order of magnitude, which can be explained by a combination of several drive-induced and Kerr-related enhancement effects as indicated by the arrows. Detailed descriptions of the individual effects are given in the main text. For a direct comparison at the photon-pressure pump power of highest cooperativity in (C), the corresponding response in the absence of drive photons (*n*_d_ = 0, red sideband power **∼**9 pW) is shown in (**E**), revealing only a small transparency window at the bottom of the undriven HF resonance. Detuning in (E) is with respect to ω_0_, and the curve was offset by **−**80 dB to match the offset of the corresponding curve in (C).

Once the HF SQUID cavity is prepared in the parametric amplifier state with *n*_d_ = 4700, we activate the photon-pressure coupling to the RF circuit by an additional pump tone on the red sideband of the signal mode, i.e., at ω_p_ = ω_s_ − Ω_0_; cf. Fig. 3A. To characterize the interaction between the driven and pumped HF mode and the RF circuit, we then detect the device response around ω_s_ with a weak third probe tone. For low sideband-pump powers, we observe a small dip inside the signal mode resonance peak (cf. Fig. 3, B and C), which gets wider and deeper with increasing pump power. The appearance of this window indicates photon-pressure–induced absorption (*57*), an effect originating in coherent driving of the RF mode by the pump-probe beating and a corresponding interference between the original probe tone and an RF-induced pump tone sideband. The width of the window in the small power regime is therefore given by the effective RF mode damping rate Γ_eff_ = Γ_0_ + Γ_pp_, where Γ_0_ = 2π · 45 kHz is the intrinsic RF circuit linewidth and Γ_pp_ is the photon-pressure dynamical backaction damping (*24*, *25*). For the largest powers, the absorption window gets shallower again, and the HF response is at the onset of normal-mode splitting, as we are approaching the photon-pressure strong-coupling regime (*24*).

For each pump power, the effective cooperativity $C_{\text{eff}}=4g_{\text{eff}}^{2}/(\kappa \Gamma_{0})$ can be determined with the RF mode linewidth Γ_0_ ≈ 2π · 45 kHz by fitting the reflection response using an effective linear mode model as discussed in the context of Fig. 2; for details, see note S8. As a result, we find that the photon-pressure cooperativity with the HF cavity signal mode is more than one order of magnitude enhanced compared to the equivalent experiment with the undriven cavity; cf. Fig. 3D. This enhancement originates from three main physical phenomena: (i) the saturation of two-level systems by the drive tone, (ii) the RF-induced flux modulation of the Kerr nonlinearity, and (iii) the intracavity amplification process arising from the presence of the strong drive. For a better understanding of the different effects, we account for all the individual contributions and indicate them as dashed lines in Fig. 3D. In the following paragraphs, we discuss them one by one.

First of all, the linewidth of the driven HF mode κ ≈ 2π · 225 kHz is reduced compared to the undriven mode (∼400 kHz), most likely due to a saturation of Two-Level Systems (TLS) by the drive tone.

From this, we get an increase in the cooperativity of ∼400/225 = 1.8, and the contribution is labeled in Fig. 3D with TLS (κ). Such a TLS saturation effect, however, is not related directly to the presence of a Kerr nonlinearity in the device and hence could be viewed as rather trivial, in contrast to the other contributions.

For the more interesting enhancement factors, which all arise from the Kerr nonlinearity in combination with the strong driving, we consider the linearized version of the multitone-driven interaction Hamiltonian.

Using $|\alpha_{d}|\gg |\gamma_{-}|,\langle \hat{c}\rangle$, where α_d_ is the drive intracavity field, γ_−_ is the sideband-pump intracavity field, and $\hat{c}$ is the intracavity probe field, the dominant contribution to the linearized interaction Hamiltonian is given by

$${\hat{H}}_{\text{int}}=\hbar [g_{0}+2g_{K}n_{d}](\gamma_{-}^{*}\hat{c}+\gamma_{-}{\hat{c}}^{†})(\hat{b}+{\hat{b}}^{†})$$ (10)

showing the usual multiphoton enhancement by the pump amplitude γ_−_ and an additional enhancement of the linearized single-photon coupling rate ${\tilde{g}}_{0}=g_{0}+2g_{K}n_{d}$ by the modulation of the Kerr constant *g*_K_. In our experiment, ∣*g*_K_ ∣ ∼ 2π · 6 Hz, ∣*g*_0_ ∣ ∼ 2π · 120 kHz, i.e., ∣*g*_K_ ∣ ≪ ∣ *g*_0_∣. The Kerr contribution to the single-photon coupling rate in the data of Fig. 3, however, is additionally enhanced by the large drive-photon number *n*_d_ ∼ 4700 and therefore contributes substantially to the total coupling rate; it increases the effective cooperativity by a factor of ∼1.9. We label this enhancement contribution in Fig. 3D with 2*g*_K_*n*_d_.

The third and final contribution to the parametrically enhanced interaction is the parametric amplification of the intracavity fields by the drive, and both the sideband pump and the probe field are amplified with different gains. Note that, in Fig. 3D, the two parts (pump and probe) are shown individually. The sideband pump field γ_−_ is far detuned from the drive, and therefore, the parametric gain at this frequency is small; the pump photon number *n*_−_ = ∣γ_−_∣^2^ is only increased by a factor of ∼1.2. Nevertheless, it is a measurable contribution, and in Fig. 3D, it is labeled with *n*_−_. The parametric amplification of the probe field $\hat{c}$ inside the driven HF resonance though is large, with an amplitude gain of G_s_ = 4. Taking into account the deep sideband-resolved limit as well as red sideband pumping and solving for the device response (see note S8), the effective multiphoton coupling rate is given by $g_{\text{eff}}=\sqrt{G_{s}n_{-}}{\tilde{g}}_{0}$ with ${\tilde{g}}_{0}=g_{0}+2g_{K}n_{d}$; i.e., the cooperativity is also enhanced by the resonance gain of the signal mode with G_s_ ≈ 4.

Using the full linearized model for calculating the theoretical device response, we find excellent agreement with the data; cf. lines in Fig. 3C. The effect of the parametric drive is not only to considerably enhance the linearized coupling rate and the cooperativity. As with increasing gain, the effective cavity resonance makes a continuous transition from an undercoupled to an overcoupled cavity (cf. Fig. 2), and also the shape of the photon-pressure–induced RF resonance inside the cavity is strongly drive dependent. For vanishing or small parametric drives, i.e., when the HF cavity still exhibits a dip in the reflection spectrum, the RF signature on the cavity lineshape resembles that of photon-pressure–induced transparency; cf. Fig. 3E. On the other hand, in the case of larger drives, i.e., when the cavity takes the shape of a peak whose resonance amplitude goes above the background, we get photon-pressure–induced absorption (*57*). Therefore, we get a highly drive-tunable system response, potentially interesting for invertible narrowband filters and microwave signal control (*58*, *59*). Additional complete datasets for *n*_d_ = 0 (G_s_ = 1) and *n*_d_ = 1650 (G_s_ ≈ 2.3) can be found in note S9.

### Enhanced RF upconversion

Photon-pressure circuits are a highly promising platform for quantum-limited sensing of RF signals by upconversion, and they are discussed in this context, e.g., for dark matter axion detection (*26*–*28*). The platform investigated here with a parametric amplifier being the RF upconverter itself might be a very interesting option toward an enhanced detection efficiency, and similar approaches have also been discussed for other Josephson-based upconversion and detection schemes (*60*–*62*).

**Fig. 4.**
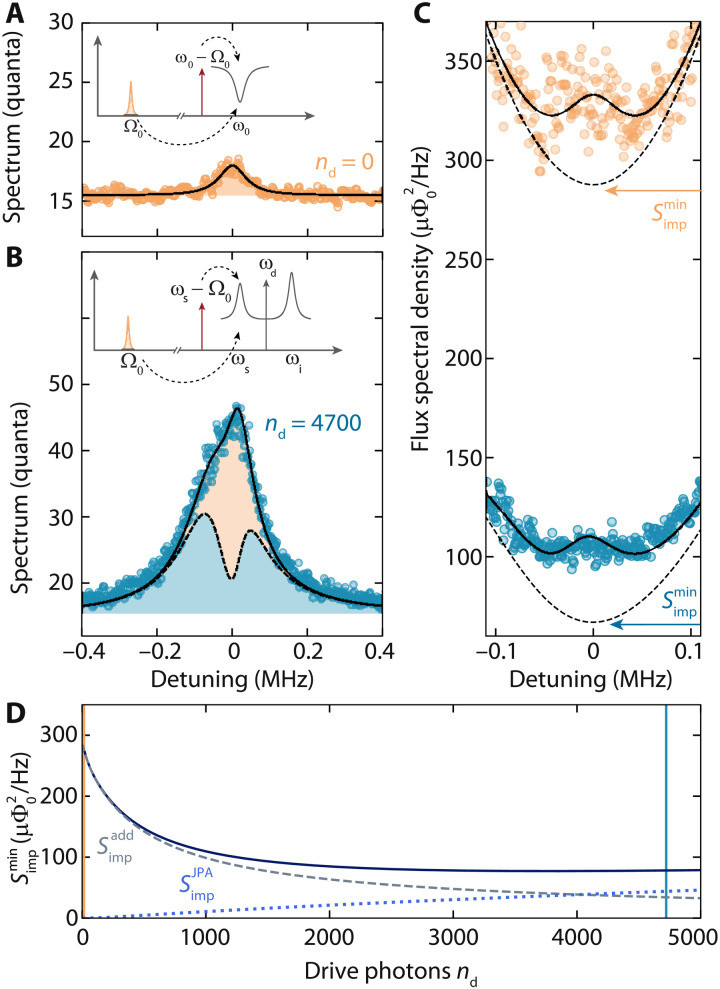
Enhanced upconversion of RF thermal noise with a photon-pressure Kerr amplifier. (**A** and **B**) Upconverted thermal noise of the RF mode, detected in the output spectrum of the SQUID cavity signal mode, for *n*_d_ = 0 and *n*_d_ = 4700, respectively. The effective cooperativity for both cases is C_eff_**≈** 0.8. Flux bias is Φ_b_/Φ_0_ = 0.48. The noise contribution from the RF mode is shaded in orange, and the blue shaded area shows the amplifier output noise for $n_{\text{th}}^{\text{RF}}=0$, which is basically amplified quantum noise with noise squashing (*4*, *67*) due to $n_{\text{th}}^{\text{RF}}<{\tilde{n}}_{\text{th}}^{\text{RF}}$, with ${\tilde{n}}_{\text{th}}^{\text{RF}}$ being the effective signal mode occupation. The detuning is given with respect to the HF cavity signal mode resonance frequency and insets show a sketch of the experimental scheme. For both data, C_eff_ ∼ 0.8. The detected noise spectra in units of quanta can be converted to RF flux spectral densities (cf. the main text), as plotted in (**C**). With internal amplification, the device RF flux sensitivity is enhanced by a factor of ∼3, and exactly on signal mode resonance, the imprecision noise by the amplifier chain is reduced by a factor of ∼3.2. The paraboloid background shape of the imprecision noise (dashed line) originates from the HF cavity susceptibility and particularly from the HF cavity linewidth being similar to the RF linewidth κ **∼** 5Γ_0_ in our device. The theoretical curve for the minimum imprecision noise versus drive photon number is shown in (**D**). Dashed and dotted lines show the individual contributions from the usual added noise (cryogenic HEMT and losses) and from the amplifier quantum noise, respectively. The two drive points from (A) to (C) are labeled with corresponding vertical lines, an orange line at *n*_d_ = 0 and a blue line at *n*_d_ = 4700.

To characterize the potential enhancement in RF flux sensitivity by the Josephson amplification in our setup, we detect the upconverted thermal fluctuations of the RF mode in the output field of the signal mode resonance with and without parametric gain; cf. Fig. 4. For this experiment, we work with a small photon-pressure cooperativity C_eff_ ≈ 0.8 for both the undriven and the amplification case to minimize the effects of dynamical backaction and mode hybridization while still having a detectable signal in the undriven case. In a direct comparison between the detected output spectrum of both setups, we observe a substantial intrinsic amplification of the upconverted RF noise in the amplification regime and, in addition, a significant background noise contribution from Josephson amplified HF cavity quantum noise; cf. Fig. 4, A and B.

In the undriven case (A), we find from the fit the residual thermal occupation of the RF mode to be $n_{\text{th}}^{\text{RF}}∼13$, i.e., a mode temperature *T*_RF_ ∼ 300 mK considerably higher than complete thermalization with the mixing chamber at *T*_b_ = 15 mK would suggest. Similar results have been observed before (*24*, *25*) and are explained by an imperfect radiation isolation between the sample and the cryogenic RF amplifier on the 3 K plate in our setup.

In the driven case (B), we obtain from the fit $n_{\text{th}}^{\text{RF}}∼15$, i.e., a mode temperature *T*_RF_ ∼ 340 mK, indicating that the RF mode seems to be slightly heated by the parametric drive. The undriven thermal HF cavity occupation is assumed to be negligible as supported by our output noise analysis in note S6, and the large Lorentzian output noise peak of the HF cavity is originating purely from parametric amplification of HF cavity quantum noise.

For a quantification of the measurement imprecision of each configuration, the detected spectrum in units of quanta *S_nn_*(Ω) is converted to RF flux spectral density using (cf. note S8)

$$S_{\Phi }^{\text{tot}}(\Omega )=\frac{2\Phi_{\text{zpf}}^{2}}{\kappa_{e}{|G(\Omega )|}^{2}{|\chi_{s}|}^{2}n_{-}g_{0}^{2}}S_{\mathrm{nn}}(\Omega )$$ (11)

$$=S_{\Phi }(\Omega )+S_{\text{imp}}(\Omega )$$ (12)

with the Josephson gain G ≠ 1 being the main difference in the prefactor to the undriven case.

The imprecision noise takes the form

$$S_{\text{imp}}(\Omega )=\frac{2\Phi_{\text{zpf}}^{2}}{\kappa_{e}{|G(\Omega )|}^{2}{|\chi_{s}|}^{2}n_{-}g_{0}^{2}}[\frac{1}{2}+n_{\text{add}}+n_{G}(\Omega )]$$ (13)

where *n*_add_ ≈ 15 is the effective noise added by the HEMT amplifier and the last term *n*_G_(Ω) is the imprecision noise contribution by the amplified quantum noise of the HF cavity.

Note that the contribution *n*_G_(Ω) to the imprecision noise is not equivalent to the added noise of a usual parametric amplifier, which would be positioned after the signal source, while, here, we have signal generation and amplification simultaneous in a single device.

In Fig. 4C, the effective RF flux spectral density is displayed, revealing a significant enhancement of the detection sensitivity in the amplifier state with ∣G∣ ∼4 at ω = ω_s_.

Because of the small ratio of linewidths in our device of κ/Γ_0_ ∼ 5, the shape of 1/∣χ_s_∣^2^ in Eq. 13 already becomes significant in a frequency window of few Γ_0_, and we see a strong frequency dependence of the imprecision noise background, which could be easily compensated for in future implementations (*62*, *63*) and which would be naturally reduced for a smaller linewidth RF mode. The minimum imprecision noise at the signal mode resonance frequency, however, is still improved by a factor of ∼3.

To evaluate the minimum imprecision depending on the flux bias point and Josephson gain, we calculate the minimum at Ω = Ω_s_ for varying drive photon numbers and obtain

$$S_{\text{imp}}^{\text{min}}=\frac{\kappa^{2}\Phi_{\text{zpf}}^{2}}{2\kappa_{e}G_{s}^{2}n_{-}g_{0}^{2}}[\frac{1}{2}+n_{\text{add}}+n_{G}(\Omega_{s})]$$ (14)

where *n*_add_ ≈ 15 and $n_{G}(\Omega_{s})=4\frac{\kappa_{e}}{\kappa }G_{s}(G_{s}-1)$. The result is shown in Fig. 4D. Note that, in the calculation of $S_{\text{imp}}^{\text{min}}$ and its individual contributions, shown in Fig. 4D, we keep the effective cooperativity and the drive frequency constant but take into account the power- and flux-dependent parameters of our device, which is equivalent to having ω_0_, ω_s_, κ, κ_e_, K, *g*_0_, and *n*_−_ change with drive photon number *n*_d_ according to the flux sweep; cf. Fig. 2. Details on the expressions and their derivations can be found in note S8.

As the drive photon number *n*_d_ is increased, the imprecision noise is significantly decreased by a factor of 3.4, due to the additional gain provided by the intracavity amplification. As the power is increased, however, eventually, the imprecision noise becomes limited by the amplification of the quantum noise of the cavity. One way to understand why the imprecision noise does not continue to improve for higher gain is that the quantum fluctuations of the cavity undergo amplification with gain $G_{s}^{2}$, while the intracavity fields from the photon-pressure coupling to the RF mode undergo only a net amplification of G_s_: The second factor G_s_ contributes instead to enhancing the cooperativity. As the intracavity gain is increased and the amplified cavity input noise begins to dominate the amplification chain of the measurement, the imprecision noise for detecting the RF fields becomes worse again as it does not undergo the same amount of amplification as the cavity input fields. Further discussions on the optimum gain for different device parameters can be found at the end of note S8, which also includes the additional references (*64*, *65*).

### Nonreciprocal bath dynamics

**Fig. 5.**
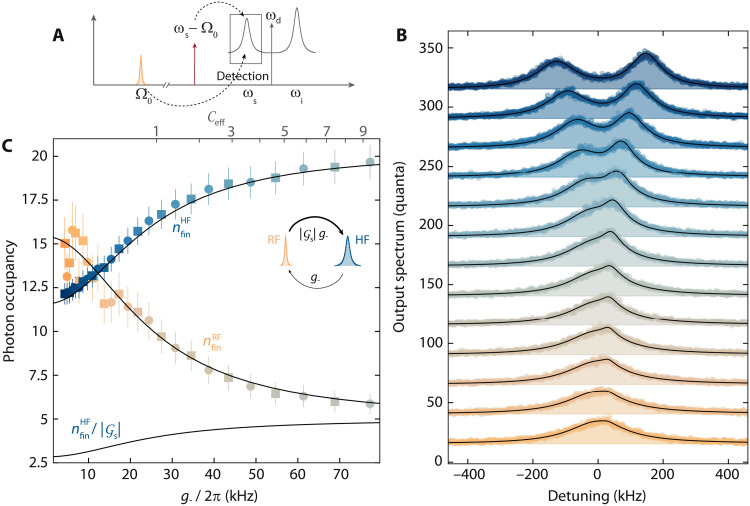
Nonreciprocal bath dynamics in sideband cooling with an amplified quantum bath. (**A**) Schematic of the experiment. The amplifier resonance (*n*_d_ = 4700, Φ_b_/Φ_0_ = 0.48) is pumped at ω_p_ = ω_s_ − Ω_0_. The output power spectrum around ω ∼ ω_s_ is detected using a spectrum analyzer. (**B**) Output spectra of the signal mode for increasing red sideband power in units of quanta (bottom curve, lowest power; top curve, largest power). Circles are data; lines and shaded areas are fits. Subsequent datasets are offset by ***+***25 each. For the lowest pump powers, the output spectrum is dominated by the amplified HF quantum noise; for medium powers, an additional peak on top of the HF noise is emerging; and for the highest powers, the two modes begin to hybridize, and the output spectrum exhibits the onset of normal-mode splitting. From the fits, we determine the resulting mode occupations, which are plotted versus $g_{-}=|\gamma_{-}|{\tilde{g}}_{0}$ and C_eff_ in (**C**). The RF occupation for *g*_−_ = 0 is $n_{\text{th}}^{\text{RF}}∼15$ and the sideband cooling reduces it to $n_{\text{cool}}^{\text{RF}}∼6$ for the largest power used. Notably, the effective HF mode occupation, arising from amplified quantum noise, is almost as high as the RF occupation for low pump powers and increases further with larger cooling of the RF mode, arriving at $n_{\text{fin}}^{\text{RF}}\approx 19\gg n_{\text{fin}}^{\text{RF}}$. This is considerably different from the usual sideband cooling, where the starting HF occupation is a fundamental limit for $n_{\text{lim}}^{\text{RF}}$ and indicates nonequilibrium heat flow from a cold to a hot reservoir. The inset sketch illustrates a nonreciprocal thermal photon conversion rate, which can be seen as the origin of the imbalanced occupation values. The effective HF photon number as seen by the RF mode is shown as a line labeled with $n_{\text{fin}}^{\text{RF}}/|G_{s}|$ where $|G_{s}|\approx 4$.

The asymmetric amplification discussed in the previous paragraph, which is limiting the improvement of the imprecision noise with gain, simultaneously leads to very unusual and nontrivial bath dynamics. To reveal this effect, we discuss what happens in sideband cooling with internal parametric gain in the HF cavity. In the high-gain regime, the effective temperature of the signal mode is in good approximation given by $T_{\text{eff}}\approx \frac{\hbar \omega_{s}}{k_{B}}{\tilde{n}}_{\text{th}}^{\text{HF}}$ with the resonance frequency of the signal mode ω_s_ and the effective mode occupation

$${\tilde{n}}_{\text{th}}^{\text{HF}}=\frac{K^{2}n_{d}^{2}}{{|\kappa +2i\Omega_{s}|}^{2}}=G_{s}(G_{s}-1)$$ (15)

arising from amplified quantum noise (*66*). At the operation point for this experiment, we get ${\tilde{n}}_{\text{th}}^{\text{HF}}\approx 12$ and *T*_eff_ ≈ 4.1 K. To investigate experimentally how this large effective occupation affects the RF mode in a sideband cooling scheme, we prepare the HF cavity again in the amplifier state by a strong drive tone and pump the signal resonance with an additional red-detuned cooling tone; cf. Fig. 5A. From the output spectrum of the driven and pumped signal resonance, which contains the amplified upconverted RF fluctuation spectral density, the RF and HF mode occupations can then be extracted; cf. Fig. 5B.

As the RF mode occupation in equilibrium, i.e., without the cooling tone, is about $n_{\text{th}}^{\text{RF}}∼15$, the naive expectation would be that considerable sideband cooling of the RF mode will not be possible in this configuration as $n_{\text{th}}^{\text{RF}}∼{\tilde{n}}_{\text{th}}^{\text{HF}}$.

The HF mode output spectra for varying power of the cooling tone (cf. Fig. 5), in combination with a theoretical analysis, however, reveal a different and unexpected scenario.

The theoretical model for the output power spectral density in units of quanta leads in good approximation to

$$\begin{array}{c} S_{\mathrm{nn}}(\omega )=\frac{1}{2}+n_{\text{add}}+\kappa_{1}\kappa {|\chi_{s}^{\text{eff}}|}^{2}\frac{{\tilde{n}}_{\text{th}}^{\text{HF}}}{G_{s}}\\ +\kappa_{1}g_{\text{eff}}^{2}{|\chi_{+}|}^{2}{|\chi_{s}^{\text{eff}}|}^{2}\Gamma_{0}n_{\text{th}}^{\text{RF}} \end{array}$$ (16)

where $\chi_{+}^{-1}=\Gamma_{0}/2+i(\Omega -\Omega_{0})$, $\chi_{s}^{-1}=\kappa /2+i(\omega -\omega_{s})$, and $\chi_{s}^{\text{eff}}=\chi_{s}/(1+g_{\text{eff}}^{2}\chi_{s}\chi_{+})$.

Note that a step-by-step derivation is presented in note S8. From the extracted occupation numbers, we find that the RF cooling factor increases with increasing power of the red sideband tone and that the RF occupation gets significantly reduced to values far below ${\tilde{n}}_{\text{th}}^{\text{HF}}$. The occupation of the HF mode simultaneously increases considerably beyond the original occupation of both modes, indicating that, even in the strong-coupling regime where the RF and HF modes fully hybridize, the populations of the two bare modes are not in balance, in stark contrast to the phenomenology of photon-pressure cooling without parametric amplification. For the largest cooling powers that we report here, the device is already slightly above the threshold for normal-mode splitting, and we obtain a final bare mode occupation of ${\tilde{n}}_{\text{th}}^{\text{HF}}\approx 19$ and ${\tilde{n}}_{\text{th}}^{\text{RF}}\approx 6$.

We note that, for the actual fits in Fig. 5B, we use a more complicated version for the power spectral density that also takes into account the frequency dependence of the parametric gain G(Ω) (eq. 147 in note S8). This more general equation allows us to reuse many of the system parameters extracted from the theoretical curves in Fig. 3C by also using the complete model, but it does not provide the intuitive insights of the approximated Eq. 16. The most important results here, the final occupation numbers $n_{\text{fin}}^{\text{RF}}\;\text{and}\;n_{\text{fin}}^{\text{HF}}$, are only slightly affected by the exact choice of the fitting equation.

The final occupation of the RF mode can be expressed in a way that resembles the cooled occupation of linear sideband cooling

$$\begin{array}{c} n_{\text{fin}}^{\text{RF}}=\frac{\Gamma_{0}}{\kappa +\Gamma_{0}}\frac{4g_{\text{eff}}^{2}+\kappa (\kappa +\Gamma_{0})}{4g_{\text{eff}}^{2}+\kappa \Gamma_{0}}n_{\text{th}}^{\text{RF}}\\ +\frac{\kappa }{\kappa +\Gamma_{0}}\frac{4g_{\text{eff}}^{2}}{4g_{\text{eff}}^{2}+\kappa \Gamma_{0}}\frac{{\tilde{n}}_{\text{th}}^{\text{HF}}}{G_{s}} \end{array}$$ (17)

Here, it is not the effective thermal occupation of the signal mode that plays a role but the effective occupation divided by the amplitude gain ${\tilde{n}}_{\text{th}}^{\text{HF}}/G_{s}$. The analog expression for the HF mode can be found in the Supplementary Materials, and there, both occupations acquire an additional factor G_s_. This suggests that, from the viewpoint of the HF mode, both modes seem hotter by G_s_.

The origin of this seemingly relative and not well-defined mode occupation values (one mode sees a “hotter” world than the other) can be found in considering the swap rates for thermal photons from one mode to the other. The heat transfer rate from the RF to the HF mode is given by G*_s_g*_−_, while the rate in opposite direction, i.e., from the HF to the RF mode, is simply given by *g*_−_; cf. eqs. 162 and 163 and the inset of Fig. 5C. The resulting nonreciprocity of thermal quanta transfer rates in our system leads to the observed flow of heat, measured in thermal photons, against an occupancy gradient in the two modes, i.e., a thermal net flow from a cold to a hot reservoir. This seeming violation of the second law of thermodynamics is a consequence of the strong driving of the imbalanced quantum system and could provide useful insights into the research field of nonequilibrium quantum thermodynamics.

## DISCUSSION

In summary, we have presented a series of experiments based on photon-pressure coupled circuits, one of which could be operated as a parametric amplifier. This operation mode leads to several interesting effects. First, the amplifier regime leads to a large parametric enhancement of the linearized single-photon coupling rate and of the photon-pressure cooperativity between the two circuits, in total up to more than an order of magnitude compared to the gainless operation. Part of this enhancement is originating from a photon-pressure modulation of the HF cavity Kerr nonlinearity, an effect described hitherto only in theoretical work. Second, we demonstrated that the internal amplification also significantly reduces the imprecision noise of upconverted RF flux signals, which is a promising perspective for optimized RF sensing applications. Last, we found that parametric amplification within the photon-pressure coupled system allows for nontrivial sideband cooling of the RF mode with a quantum-heated amplifier, where the effective, quantum noise–related temperature of the amplifier mode is not constituting the cooling limit for the RF mode. Furthermore, we have shown that Kerr amplification in the photon-pressure cavity leads to unexpected bath dynamics that, if further explored, could potentially lead to interesting applications in quantum bath engineering.

Our experiments reveal that Kerr nonlinearities can be an extremely versatile and useful resource for engineering enhanced and novel photon-pressure based devices. We believe that the investigation of the possibilities has just begun, and a fruitful exchange of ideas and protocols with closely related platforms such as Kerr optomechanics will advance the exploration of nonlinearities in these systems further. The Josephson-based Kerr nonlinearity has also already been demonstrated to allow for a variety of interesting microwave photon manipulation techniques such as cat-state generation and stabilization, bosonic code quantum information processing, nonreciprocal photon transport, or the implementation of superconducting qubits. Integrating some of these possibilities into photon-pressure or Kerr optomechanical platforms might allow for elaborate quantum control of RF circuits and mechanical oscillators in the future.

## MATERIALS AND METHODS

The photon-pressure system used in this experiment consists of two galvanically connected lumped-element LC circuits, and it was engineered via a multilayer nanofabrication process. The HF mode of the circuit comprises two interdigitated capacitors, a SQUID containing two Josephson junctions and two linear inductors. Aside from the firstly patterned 50-nm-wide, 100-nm-long, and 15-nm-thick aluminum nanobridge junctions and their respective 500 × 500 nm^2^ contact pads, the circuit is made of a ∼70-nm-thick aluminum layer on a silicon substrate.

Both layers were patterned via a sputtering deposition in combination with electron beam lithography and liftoff. In addition, an argon milling process (∼2 min) was performed in situ before the second deposition step to provide good electrical contact between the two layers. The RF mode is formed by a parallel plate capacitor with a ∼130-nm-thick amorphous silicon layer as dielectric and a short inductor wire, which simultaneously acts as the loop of the SQUID. The inductor wire was patterned together with the bottom capacitor plate and the HF circuit mode components. Subsequent to this step, the dielectric deposition takes place, i.e., a PECVD (plasma-enhanced chemical vapor deposition) process followed by a reactive ion etching step and O_2_ plasma ashing. Last, we patterned the top capacitor plate. This one is made of a 250-nm aluminum layer, and it was fabricated via another sputtering-liftoff procedure, which once again included an in situ argon milling to guarantee good contact between the plates. A step-by-step description of the device fabrication is given in note S1.

## Supplementary Materials

### This PDF file includes:

Notes S1 to S9 Fig. S1 to S11
